# Toward Fast Screening of Organic Solar Cell Blends

**DOI:** 10.1002/advs.202000960

**Published:** 2020-06-18

**Authors:** Artem Levitsky, Giovanni Maria Matrone, Aditi Khirbat, Ilaria Bargigia, Xiaolei Chu, Oded Nahor, Tamar Segal‐Peretz, Adam J. Moulé, Lee J. Richter, Carlos Silva, Natalie Stingelin, Gitti L. Frey

**Affiliations:** ^1^ Department of Material Science and Engineering Technion—Israel Institute of Technology Haifa 3200003 Israel; ^2^ Department of Materials and Centre of Plastic Electronics Imperial College London London SW7 2AZ UK; ^3^ School of Materials Science and Engineering Georgia Institute of Technology Atlanta GA 30332 USA; ^4^ School of Chemistry and Biochemistry Georgia Institute of Technology Atlanta GA 30332 USA; ^5^ Department of Chemical Engineering University of California Davis CA 95616 USA; ^6^ Department of Chemical Engineering Technion—Israel Institute of Technology Haifa 3200003 Israel; ^7^ Materials Science and Engineering Division National Institute of Standards and Technology Gaithersburg MD 20899 USA; ^8^ School of Physics Georgia Institute of Technology Atlanta GA 30332 USA

**Keywords:** bulk heterojunctions, morphology, organic electronics, photovoltaic devices, screening

## Abstract

The ever increasing library of materials systems developed for organic solar‐cells, including highly promising non‐fullerene acceptors and new, high‐efficiency donor polymers, demands the development of methodologies that i) allow fast screening of a large number of donor:acceptor combinations prior to device fabrication and ii) permit rapid elucidation of how processing affects the final morphology/microstructure of the device active layers. Efficient, fast screening will ensure that important materials combinations are not missed; it will accelerate the technological development of this alternative solar‐cell platform toward larger‐area production; and it will permit understanding of the structural changes that may occur in the active layer over time. Using the relatively high‐efficiency poly[(5,6‐difluoro‐2,1,3‐benzothiadiazol‐4,7‐diyl)‐alt‐(3,3′′′‐di(2‐octyldodecyl)‐2,2′;5′,2′′;5′′,2′′′‐quaterthiophen‐5,5′′′‐diyl)] (PCE11):phenyl‐C61‐butyric acid‐methyl‐ester acceptor (PCBM) blend systems, it is demonstrated that by means of straight‐forward thermal analysis, vapor‐phase‐infiltration imaging, and transient‐absorption spectroscopy, various blend compositions and processing methodologies can be rapidly screened, information on promising combinations can be obtained, reliability issues with respect to reproducibility of thin‐film formation can be identified, and insights into how processing aids, such as nucleating agents, affect structure formation, can be gained.

## Graphical abstract

Identifying high‐performing organic solar cell blends is still an exhausting process relying on labor and time intensive trial‐and‐error procedures. It is shown that a combination of three simple and abundant techniques, thermal analysis, vapor‐phase‐infiltration imaging, and transient‐absorption spectroscopy, can provide the multiscale materials and processing insights that are sufficient to guide the fast screening of organic solar cell blends.

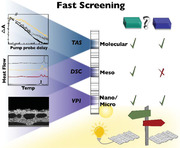

Organic solar cells (OSCs), which are generally based on bulk heterojunctions (BHJ) between an electron donor and an electron acceptor,^[^ ^1^ ^]^ have recently achieved power‐conversion efficiencies (PCE) over 16%,^[^ ^2^ ^]^ rendering this technology attractive for commercial exploitation. A significant contribution to this progress resulted from the advancement of new materials. This has led to a large number of novel OSC materials and, in turn, a plethora of donor:acceptor combinations.^[^ ^3^ ^]^ However, since the device performance is interlinked in a complex way not only with the properties of the individual components but also with the blends’ microstructure and phase morphology (e.g., the length scale of the donor:acceptor phase separation; the donor:acceptor interfacial area; their phase purity; and the formation of bi‐continuous percolation paths),^[^ ^4^, ^5^, ^6^, ^7^ ^]^ identifying promising donor:acceptor combinations is still an exhausting process relying on tedious trial‐and‐error procedures. Such an approach is unsustainable, especially considering the large number of possible OSC blend combinations.

Currently used methodologies for materials screening generally rely on the fabrication of many sets of devices and analysis of large libraries of data using statistical and machine learning algorithms.^[^ ^8^, ^9^, ^10^ ^]^ While useful for parameter optimization, these approaches can be fundamentally sophisticated and often do not provide the direct scientific insights that are easily translated toward the design and synthesis of new materials, nor assist with materials selection and/or the choice of processing conditions. Here we demonstrate that a minimum set of three, simple, and abundant characterization tools, differential scanning calorimetry (DSC), vapor phase infiltration (VPI), and transient absorption spectroscopy (TAS), provides sufficient multiscale information to guide the fast screening of organic solar cell blends—a task that these techniques on their own, or combined with other structural characterization techniques such as X‐ray diffraction or transmission electron microscopy, cannot deliver.

To illustrate the usefulness of our screening method, we selected poly[(5,6‐difluoro‐2,1,3‐benzothiadiazol‐4,7‐diyl)‐alt‐(3,3′′′‐di(2‐octyldodecyl)‐2,2′;5′,2′′;5′′,2′′′‐quaterthiophen‐5,5′′′‐diyl)] (PCE11)^[^ ^11^ ^]^ and phenyl‐C_61_‐butyric‐acid‐methyl ester (PCBM) as a model donor:acceptor system. Their chemical structures are displayed in **Figure** **1**a. We scrutinize our methodology both on spin‐coated and more scalable wire‐bar‐coated PCE11:PCBM blend films/devices, with and without processing additives such as nucleating agents, and highlight why up‐scaling can be a difficult task if rapid feedback is not provided.

**Figure 1:**
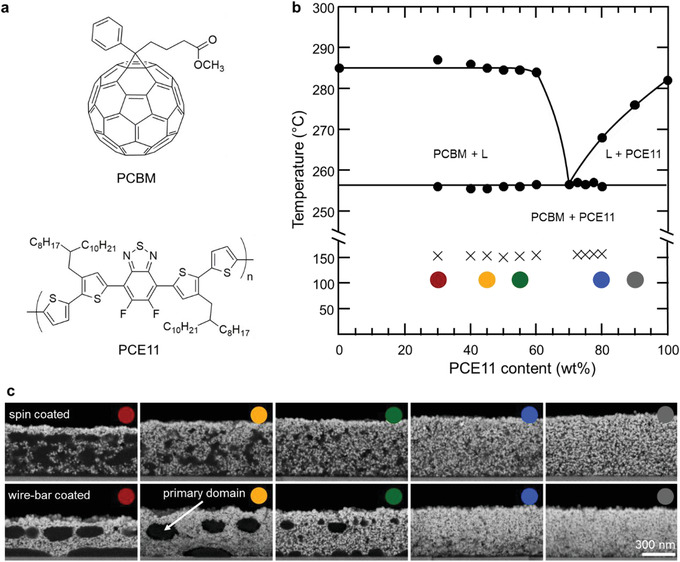
Phase behavior and solid state microstructure of PCE11:PCBM blend films. a) Chemical structures of PCBM and PCE11. b) Nonequilibrium phase diagram of the PCE11:PCBM system deduced from DSC first heating thermograms, obtained at scan rate of 10 °C min^−1^, of high‐temperature drop‐cast samples. A eutectic behavior is found with a eutectic composition around 75–80 wt% PCE11 and a eutectic temperature around 258 °C. The black crosses represent the cold‐crystallization temperatures, as deduced by a line drawn tangential to the thermograms to intercept the onset of the heat‐flow slope variation. c) Cross‐section HRSEM BSE micrographs of spin‐coated (top row) and wire‐bar coated (bottom row) PCE11:PCBM films of different blend compositions, after exposure to 80 cycles of DEZ and water at 60 °C, showing selective deposition of ZnO in the PCE11‐rich domains (bright contrast). In contrast, PCBM‐rich domains inhibit ZnO deposition, and, thus, are characterized by dark contrast. The selective “staining” of PCE11‐rich domains by ZnO in the VPI process effectively maps the phase separation as well as the size‐ and spatial distribution of the donor‐ and acceptor‐rich domains in PCE11:PCBM blend films. The scale bar for all the micrographs is 300 nm.

We start our discussion with information that can be obtained from DSC, comparing the thermal behavior of films drop‐cast at room temperature and 120 °C, respectively, for both the neat materials (PCE11, PCBM) and blends of different compositions. The two sets of thermograms are displayed in Figure S1, Supporting Information. From these, we conclude that both sample sets feature a very similar eutectic behavior with the eutectic composition being around 75–80% mass fraction (wt%) PCE11 and the eutectic temperature around 258 °C (see Figure 1b for an illustration of the phase diagram plotted based on the thermograms of the high‐temperature‐cast samples). While various donor:acceptor systems have been reported to display an eutectic phase behavior, here, we use the comparison of the individual thermograms of the differently cast films to obtain information of how the selected processing conditions affect the samples‘ solid‐state microstructure formation with the goal to provide predictive insights on the solidification tendencies of these OSC bends.

More specifically, we concentrate on the relative enthalpies of fusion for the fullerene, Δ*H*_f_^relative^ (PCBM), with respect to the Δ*H*_f_ of neat PCBM films. We find that high‐temperature‐cast films display notably smaller Δ*H*_f_^relative^ (PCBM) compared to blends deposited at room temperature (≈46% and ≈74%, respectively, e.g. for blends of 30 wt% PCE11; Figure S1, Supporting Information). One reason for this observation is that high‐temperature casting leads to higher molecular order in neat PCBM films, as evidenced by the higher enthalpy of fusion found in these samples compared to neat PCBM cast at room temperature. This can be expected based on, for example, an increased diffusion when casting at higher temperatures helping molecules to order. However, in blends, we do not observe this effect. We, thus, conclude that the high‐temperature‐cast conditions may lead in the blend solutions to a higher compatibility between the donor and acceptor molecules. Upon solvent evaporation, this would—in blends—limit the improvement that can be achieved in molecular ordering compared to the neat films. This effect can be expected to lead to some vitrification (glass‐formation) of the fullerene‐rich domains when selecting conditions that favor rapid film formation.

Since the phase diagrams established by DSC are deduced for nonequilibrium structures, as the above examples highlight (see also refs. [12, 13]), they are not always straight‐forward to interpret. Also, they can only be constructed for material obtained from relatively thick films. For screening purposes, we therefore needed complementary methodologies that allow assessment of thin, device‐relevant films. We opted for VPI as it can directly visualize the phase behavior and nonequilibrium phase morphology of device‐relevant OSC blend thin films, focusing on PCE11:PCBM BHJ structures as well as neat PCE11 and PCBM films. In a first set of experiments, we processed these systems via spin‐coating from hot solutions (130 °C) preheating the spin‐coating chuck and the substrates to 130 °C. To ensure comparison with device performances, all films were prepared to be ≈300 nm thick. The films were left to dry overnight in inert atmosphere and then were exposed to gaseous metal‐oxide precursors, in this case diethyl zinc (DEZ) and water, followed by in situ conversion to the metal oxide^[^ ^14^, ^15^, ^16^, ^17^, ^18^ ^]^ as outlined in Figure S2, Supporting Information.

Cross‐section high‐resolution scanning electron microscopy (HRSEM) back‐scattered electron (BSE) images of the neat PCE11 and PCBM films after VPI show that the precursor can only diffuse through the polymer phase (Figure S2, Supporting Information). Indeed, we find homogenous, rich sub‐surface deposition of ZnO within neat PCE11 films, leading to a bright contrast; no ZnO is deposited within PCBM films, resulting in a dark contrast even after prolonged exposure times, in agreement with our previous observations.^[^ ^14^, ^15^, ^16^ ^]^

In blends, the z‐contrast between inorganic ZnO deposits in PCE11‐rich domains and domains where the presence of PCBM inhibits ZnO deposition, enables rapid analysis of the BHJ phase morphology as a function of composition and processing conditions. Figure 1c (top row) shows cross‐section HRSEM BSE micrographs of spin‐coated PCE11:PCBM films of different compositions. The films with high PCE11 content, that is, 80–90 wt% PCE11, feature homogeneous, sub‐surface ZnO deposition (Figure 1c) similar to what is found for neat PCE11 (Figure S2, Supporting Information). This is in agreement with DSC (Figure 1b), which predicts these blends to be predominantly composed of eutectic morphologies (i.e., finely‐phase separated PCE11:PCBM regions) with some PCE11‐rich domains. When decreasing the PCE11 content below 80 wt%, we observe the evolution of distinct, seemingly interconnected PCBM‐rich domains that increase in size with increasing PCBM content (Figure 1c, top row) as also expected from DSC. We attribute these domains based on classical materials nomenclature to be primary PCBM regions, where “primary” means that they are formed prior to the eutectic transition.

Based on the phase morphology only, slightly hypoeutectic films (i.e., films with compositions slightly‐off eutectic toward the fullerene‐rich side) are expected to perform best as active solar cell layers^[^ ^12^, ^13^ ^]^ as they feature interconnected, fullerene‐rich domains that are of limited size and, thus, should be able to support exciton dissociation while also assisting charge extraction. However, other structural features, including phase purity of domains, degree of molecular order, and crystalline quality, will play important roles in the photovoltaic energy‐conversion process. Elucidation of these requires, however, the collective information from a broad combination of experimental tools including grazing‐incidence wide‐angle X‐ray diffraction and soft‐X‐ray synchrotron methodologies that are not compatible with fast screening. We, thus, selected to carry out TAS to obtain rapid, additional information on PCE11:PCBM thin films. With current ultrafast laser technology, TAS allows one to examine at least 10 samples per day and can provide the necessary critical information on population dynamics with sub‐ps time resolution over nanosecond time windows. Since these processes are intricately interlinked with the very local electronic landscape of OSC blends that is dictated by local structural features, such as donor chain conformation, molecular interfaces, and local fullerene packing, TAS is very complementary to the information revealed by VPI and thermal analysis, and helps us come to an informed decision during materials screening.

Data obtained from TAS measurements as a function of pump‐probe delay, performed on neat films and blends deposited via spin‐coating, are presented in **Figure** **2**a (top panel) and Figure S3, Supporting Information. All spectra show a ground‐state bleaching signal in the 500–690 nm range and a photo‐induced absorption (PA) feature at 720–890 nm (Figure S3, Supporting Information) that, by comparison to previous studies, can be ascribed to the absorption of photo‐generated polarons.^[^ ^19^ ^]^ The spectra at early times in all blends loosely resemble those of the neat films, with slightly hypoeutectic blends (45–55 wt% PCE11) displaying a higher PA signal (Figure 2a, top panel). Moreover, the different blends show a dynamic behavior that depends on film composition, with a higher net density of charges (longer‐lived PA signal) over long timescales found for blends of 45–55 wt% PCE11.

**Figure 2:**
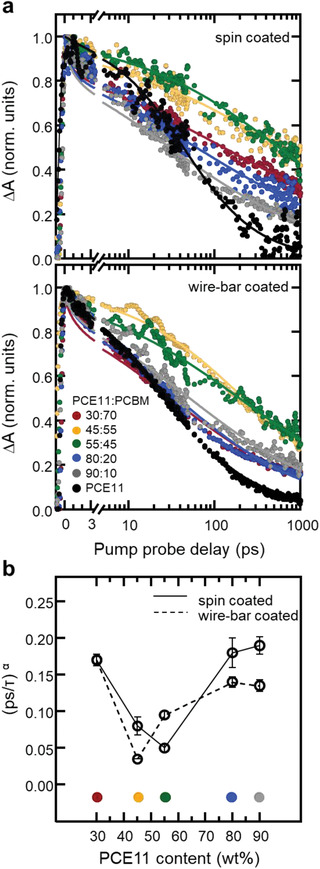
Transient absorption spectroscopy dynamics of the PCE11:PCBM system. a) Transient absorption dynamics at probe wavelength of 820 nm for the six selected PCE11:PCBM blend composition films, spin coated (top panel) and wire‐bar coated (bottom panel). The solid lines represent the fit of the data to an asymptotic power law. b) Fitting the dynamics to an asymptotic power law function *S*(*t*)∝1/(1 + (*t*/*τ*)^*α*^) allows one to retrieve a (*ps*/*τ*)*^*α*^* decay parameter. (*ps*/*τ*)*^*α*^* as a function of PCE11 content with the error bars representing the upper and lower 95% confidence intervals of the nonlinear fit. (*ps*/*τ*)*^*α*^* represents the average recombination rate of polarons over the timescale of the experiment. This means that the higher this parameter is, the faster, on average, is the decay of the charges.

Considering the complex nature of the PA‐band dynamics and the fact that a continuous distribution of decay times is necessary to account for its temporal behavior, we fitted the data to an asymptotic power‐law function, *S*(*t*)∝1/(1 + (*t*/*τ*)^*α*^), phenomenologically describing a decay function with a distribution of rates (see Figure S3, Supporting Information, or Experimental Section).^[^ ^20^, ^21^ ^]^ From the fitting we retrieve a normalized decay parameter ([*ps*/*τ*]*^*α*^*), which is representative of the average polaron recombination rate over the timescale of the experiment: an increase in the decay parameter is associated with decreased charge lifetime.

We observe that (*ps*/*τ*)*^*α*^* starts around ≈0.2 for hypereutectic and close‐to‐eutectic compositions (80 and 90 wt% PCE11; Figure 2b). It reaches a minimum of ≈0.045 for slightly hypoeutectic blends with small, interconnected PCBM domains (55 wt% PCE11; Figure 1c, top row). For PCBM‐rich blends (45 wt% PCE11), (*ps*/*τ*)*^*α*^* increases to ≈0.085, reaching ≈0.175 for blends of 30 wt% PCE11 (Figure 2b). Taking into account the slow average decay of charges according to the (*ps*/*τ*)*^*α*^* deduced from TAS, and the finely phase‐separated morphology with homogenously distributed small PCBM domains observed in VPI (average diameter of 35 nm; Figure 1c), we predict best device performances for blends with a composition around 55 wt% PCE11.

The combination of DSC, VPI, and TAS is sufficient to screen donor:acceptor blends to identify suitable systems for solar cells prior to device fabrication; it does not require devices to be made. Yet, to confirm the above prediction we produced devices using identical processing conditions used for VPI (**Figure** **3**a). The best devices are found for spin‐coated blends of 55 and 45 wt% PCE11, in agreement with the literature.^[^ ^11^, ^22^ ^]^ Somewhat surprisingly, OSCs even with relatively high PCBM content (30 wt% PCE11) still displayed decent performances, while close‐to‐eutectic and hypereutectic mixtures without PCBM‐rich domains and high (*ps*/*τ*)*^*α*^* values feature low short‐circuit currents, *J*_sc_, and low fill factors, FF (Figure 3b). These observations are in accordance with the picture that small, interconnected, and relatively phase‐pure acceptor domains can act as wells for the generated charges relative to amorphous or mixed domains, and/or provide an entropic (carrier‐density gradient) driving force due to formation of the carriers in a finely intermixed phase.^[^ ^23^, ^24^ ^]^

**Figure 3:**
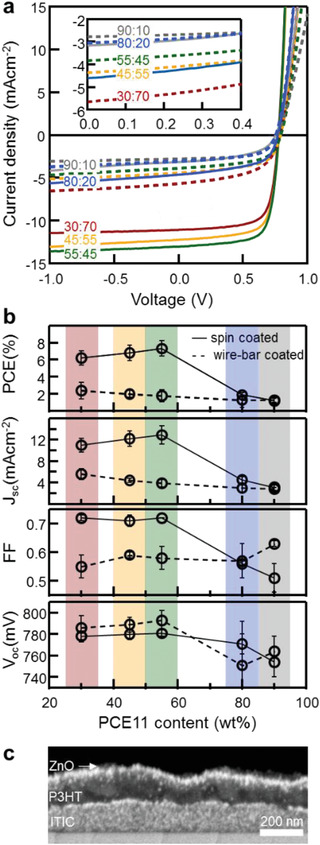
Comparison of OSC performance between different PCE11:PCBM blend compositions, spin‐coated and wire‐bar coated. a) *J*–*V* characteristics acquired at 100 mW cm^−2^ AM1.5G irradiation of PCE11:PCBM OSCs having the following structure: ITO/ZnO nanoparticles/PCE11:PCBM BHJ/MoO_3_/Al, deposited from different blend compositions (indicated by PCE11:PCBM ratios), prepared by spin coating (solid lines) and wire‐bar coating (dashed lines). The inset highlights the characteristics of low‐performing devices. b) Photovoltaic parameters: PCE, *J*_SC_, FF, and *V*_OC_, extracted from the *J*–*V* curves in as a function of blend composition and deposition technique. Error bars represent the standard deviation from measurement of a minimum of 11 devices. c) Cross‐section BSE HRSEM images of a poly(3‐hexylthiophene‐2,5‐diyl) (P3HT)/3,9‐bis(2‐methylene‐(3‐(1,1‐dicyanomethylene)‐indanone))‐5,5,11,11‐tetrakis(4‐hexylphenyl)‐dithieno[2,3‐d:2′,3′‐d′]‐s‐indaceno[1,2‐b:5,6‐b′]dithiophene (ITIC) bilayer on a Si substrate. Selected VPI conditions lead to ZnO nucleation and growth mainly inside the ITIC film, resulting in bright contrast (bottom layer); while the P3HT film under these conditions does not maintain the precursors resulting in dark contrast (top layer).

To contrast with the well investigated spin‐coated PCE11:PCBM blends, we applied our fast screening methodology to films produced via wire‐bar coating, which is often used to scrutinize up‐scaling options. Hot solutions were deposited on substrates of 120 °C. VPI dramatically reveals the effect the different processing has on the overall phase morphology (Figure 1c, bottom row). While the hot wire‐bar coated blends follow again a clear eutectic behavior, the primary PCBM domains, visualized by VPI, are drastically more developed and significantly larger in size compared to their spin‐coated counterparts. More specifically, blends of 45 wt% of PCE11 comprise large, sphere‐like PCBM domains of ≈140 nm in size, positioned in a relatively periodic pattern along rows. In addition, a strong thickness dependence is found (Figure S4, Supporting Information) with the fullerene vertically phase‐separating to the bottom interface at high PCBM content—observations that are reminiscent of structures formed via spinodal decomposition. Furthermore, the dependency of (*ps*/*τ*)*^*α*^* with composition is shifted for wire‐bar coated films with respect to spin‐coated structures (Figure 2a, bottom panel). For close‐to‐eutectic and hypereutectic compositions, that is, 80–90 wt% PCE11, (*ps*/*τ*)*^*α*^* is somewhat lower (≈0.14) than for spin‐coated films (Figure 2b), while it reaches ≈0.17 for highly hypoeutectic compositions (30 wt% PCE11). However, (*ps*/*τ*)*^*α*^* reaches a minimum (≈0.03) only for highly hypoeutectic blends comprising 45 wt% PCE11 (Figure 2b) where relatively large PCBM‐domains are observed. A possible reason for the observed TAS results is that only in this composition regime the fullerene domains become sufficiently phase pure/molecularly ordered. While we could not assess this directly on wire‐bar coated films, we do find for blends with higher PCE11 content (≈55 wt% PCE11), drop‐cast at 120 °C, very low Δ*H*_f_ (PCBM) (≈10% of the Δ*H*_f_ value of neat PCBM films; Figure S1, Supporting Information), indicating vitrification.^[^ ^25^ ^]^ This effect can be expected to be similar or more pronounced for the hot wire‐bar coated films. We thus predict that in the case of wire‐bar coated BHJs, analogous to previously‐reported systems with non‐fullerene acceptors,^[^ ^13^ ^]^ best device performances will not be in the slightly hypoeutectic regime but at higher acceptor content where we find higher fullerene enthalpies in high‐temperature drop‐cast films, leading to slower dynamics.

Consistent with the poor morphology visualized by the VPI, the wire‐bar devices underperform the spun‐coated devices. The best device performance for wire‐bar coated films is observed for devices of 45 and 30 wt% PCE11. As with the spun‐coated films, the 30 wt% PCE11 outperform expectation based on TAS alone, indicating that TAS, while useful for realizing charge photo‐generation, cannot solely screen OSC blends. VPI provides information that is complimentary to TAS showing vertical phase separation with PCBM forming large, flat domains at the electron‐collecting electrode (Figure 1c, bottom row). We attribute the wire bar coated device performances to the presence of this PCBM‐rich layer which is favorable when using inverted device architectures, as done here. However, we also note that reliance on such spontaneous vertical phase separation can lead to reproducibility issues as this process is very sensitive to deposition conditions, nature of substrate, and film thickness (Figure S4, Supporting Information).

We note, additionally, that in hot wire‐bar coated devices *J*_sc_ is not very composition dependent, slightly increasing only in PCBM‐rich blends (Figure 3b). This suggests that these devices are dominated by the eutectic PCBM:PCE11 regions that are finely phase‐separated, with only limited influence from the primary PCBM domains that form and can be seen in VPI. This leads to low device performance. In contrast, for spin‐coated blends, *J*_sc_ and FF increase in an abrupt fashion as soon as small primary PCBM domains are observed (Figure 3b), supporting the idea that one of the key design requirements of materials for BHJ OSCs is their tendency to form phase‐pure, molecularly ordered domains (e.g., in the form of primary acceptor regions). These domains function as energetic sinks for photo‐generated charge carriers relative to amorphous or mixed domains, thereby, enabling the spatial separation of these photo‐generated charges and assisting with charge extraction.^[^ ^26^ ^]^

In order to test this view and further exploit the relative simplicity of our ‘screening’ method, we produced films via wire‐bar coating at 120 °C using commercially available nucleating agents during processing, exploring whether this will limit vitrificaiton effects due to high‐temperature casting and, thus, will lead to smaller, more ordered primary domains and, in turn, to an improved device performance. These additives, frequently employed to control the solidification of bulk commodity polymers, have been demonstrated to aid the structure development of a large number of organic semiconductors, polymeric and small molecular, when processed from the melt, solution, or vitreous solid state.^[^ ^27^ ^]^ We used 1,3:2,4‐di‐o‐methylbenzylidene‐*D*‐sorbitol (MDBS) as the nucleating agent^[^ ^28^ ^]^ following similar procedures as reported previously.^[^ ^27^, ^28^ ^]^

The impact of the additive on the structure formation of as‐cast films can be readily deduced from the DSC data presented in Figure S5, Supporting Information, showing three sets of thermograms for films drop‐cast at room temperature and 120 °C, pristine and with nucleating agent. Intriguingly, the nucleating agent has strongest impact on the solidification of the PCE11 and PCBM in the eutectic domains but not the primary domains. Wire‐bar‐coated films cast at 120 °C with nucleating agents feature significantly higher enthalpies for the endotherms attributed to the eutectic temperature, compared to high‐temperature‐cast films where no additives were used (Figure S5 and Table S1, Supporting Information). The primary PCBM domains become, in contrast, less crystalline/ordered, as deduced from the lower fullerene‐melting enthalpies we measure for nucleated structures. These observations are in accord with VPI where we find no drastic changes in domain size/distribution for the primary PCBM regions in nucleated samples compared to blends where no nucleating agent was added (Figure S5, Supporting Information), implying that the nucleating agents assist the slowest structure‐formation process, here the eutectic decomposition, which is a diffusion‐limited process—that is, the eutectic PCE11:PCBM regions become more crystalline but not the primary regions.

The increased degree of crystallinity of eutectic PCE11/PCBM phases, combined with the reduction of the molecular order of the primary PCBM domains, has a direct effect on the dynamics of the photo‐generated charges. (*ps*/*τ*)*^*α*^* becomes notably less dependent on blend composition when films were produced with addition of nucleating agents, with its values merging toward the one deduced for neat PCE11 and blends close to the eutectic composition, that is, systems with fast exciton decays (Figure S6, Supporting Information). Accordingly, it is not surprising that corresponding devices are of very low efficiencies (not shown). This observation, hence, supports the view that primary PCBM domains need not only to be present in OSC blends but also need to be molecularly ordered to limit charge recombination in addition to providing charge‐extraction pathways and energy sinks assisting charge generation. Using high‐content fullerene blends to achieve this is, though, not a valid option. The thickness‐dependence of the structure formation of wire‐bar‐coated films (Figures S4 and S5, Supporting Information) leads to large variations in the charge‐carrier dynamics and, thus, a large sample‐to‐sample variation (Figure S6, Supporting Information). This is somewhat reduced using nucleating agents; but at the expense of inducing fast charge‐decay dynamics.

Additional techniques could be added to the three screening methods used here to provide a more detailed picture. Automated sample handlers, for instance, now allow grazing‐incidence small‐angle and wide‐angle scattering (GI‐SAXS and GI‐WAXS, respectively) to be performed with throughput in excess of 100 films per day, and could be incorporated into a screening paradigm. However, the limited q‐range of hard X‐ray GI‐SAXS (only the spin‐coated films comprising 30 wt% PCE11 show a distinct feature in GI‐SAXS, Figure S7, Supporting Information) compared to the wide range of feature sizes (Figure 1c) can limit its general utility. Similarly, GI‐WAXS provides important information on the degree of crystallinity and texture; however, these do not unambiguously correlate with performance, as diverse morphologies can produce similar device performances.^[^ ^29^ ^]^

High‐resolution transmission electron microscopy (HRTEM) imaging and, specifically, tomography, can provide unprecedented detail on the 3D connectivity of domains. **Figure** **4** shows 3D‐morphology reconstructions of two samples: a spin‐coated film comprising 45 wt% PCE11 (Figure 4a) and a wire‐bar coated film with 30 wt% PCE11 (Figure 4b) based on three perpendicular cross‐section images that are frames from movies showing the full 3D‐morphology reconstruction of the selected sample volume (Movies S1 and S2, Supporting Information). The images validate the insights obtained from the 2D‐HRSEM images (Figure 1c) that the 45 wt% PCE11 spin‐coated film architecture is based on interconnected donor:acceptor networks, while the wire‐bar coated film of 30 wt% PCE11 is composed of coarse, isolated PCBM‐rich domains within a PCE11‐rich matrix. This picture is supported by the tomography data representing the 3D donor:acceptor interface (Figure 4c). Unambiguously, while very helpful, grazing‐incidence scattering and HRTEM are best applied to detailed studies of optimal devices—but not fast screening. In contrast, the VPI/DSC/TAS‐screening can be executed relatively fast, using standard lab techniques and using a range of systems, from neat films to blends of different composition/thicknesses, promising wide‐applicability, especially to new OSC‐blend combinations.

**Figure 4:**
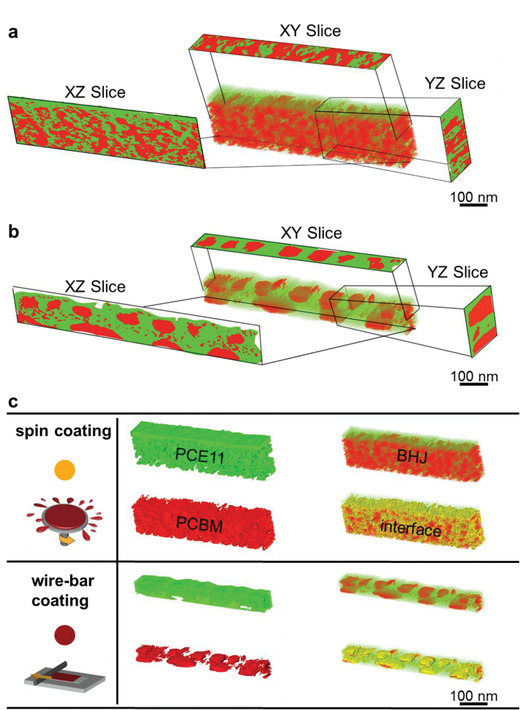
Tomographic reconstruction of selected PCE11:PCBM BHJs films from TEM and STEM tilt‐series showing the 3D‐spatial distribution of PCE11‐rich domains (green), PCBM‐rich domains (red), and the interface between them (yellow). a) Three perpendicular cross‐section images that are frames from a movie showing the full 3D‐morphology reconstruction of the 45 wt% PCE11 film prepared by spin coating. See Movie S1, Supporting Information. b) Three perpendicular cross‐section images that are frames from a movie showing the full 3D‐morphology reconstruction of the 30 wt% PCE11 film prepared by wire‐bar coating. See Movie S2, Supporting Information. The BHJ morphologies revealed by the slices (*XZ* and *YZ*) perpendicular to the film plane are in full agreement with the HRSEM micrographs of the same blends shown in Figure 1c confirming VPI as a fast and useful technique for BHJ morphology screening. c) Separated reconstruction of PCE11‐rich domains (green), PCBM‐rich domains (red), and the interface (yellow) between them. The additional dimension revealed by tomography clearly demonstrates that the spin coating leads to large interfacial area between the PCE11‐PCBM domains (yellow), together with a branched structure of the two phases. In contrast, the morphology of wire‐bar coated films is coarse with large, separated PCBM‐rich domains (red) embedded in a thin PCE11‐rich film (green).

In conclusion, we demonstrated a fast‐screening methodology that employs a combination of thermal analysis, vapor infiltration, and transient absorption spectroscopy to deliver the minimum multiscale information on donor:acceptor blend films needed to make informed decisions on BHJ thin films prior to device fabrication and device optimization. The advantage of our approach lies in its simplicity and its broad application, with all three methodologies being readily available and allowing multiple samples to be measured per day. In the screening workflow, thermal analysis establishes the target composition range and undesirable processing effects, such as over‐vitrification. VPI performed on films of compositions identified in thermal analysis provides information, for example, on processing “dead ends” including large‐scale phase separation and/or lack of domain connectivity; while TAS screens blends of VPI‐selected compositions and processed via DSC‐identified protocols with respect to their photo‐physical behavior, including their local recombination kinetics. Importantly, this workflow should also be applicable to blends comprising non‐fullerene acceptors. Initial VPI experiments, for instance, show that a contrast between donor and acceptor domains can be realized (Figure 3c), however, with the contrast inversed to that in PCE11:PCBM blends. This inverse contrast provides another handle for selective domain “staining” to directly visualize the phase behavior and phase morphology of blends comprising non‐fullerene acceptors—information that will be highly useful to understand the complex phase behavior of many non‐fullerene acceptors. Finally, we like to also mention that our methodology, most promising to deliver “Go/No‐Go” decisions on materials combinations/processing conditions used prior to device fabrication, could also be used on complete device structures, for instance, to provide rapid feedback on the origins of device degradation over time (e.g., the burn‐in caused by morphology changes).^[^ ^22^ ^]^ Figure S8, Supporting Information, shows data for VPI performed on completed devices. In case of TAS, measurements would need to be performed outside the electrode area, while for thermal analysis fast calorimetry methodologies are required, which in principal allow measurements on devices—provided electrodes and interlayers feature distinct thermal transitions outside the temperature regime of interest. Our approach, thus, promises to deliver significant insights beyond screening, assisting materials design for the robust processing of OSCs.

## Experimental Section

##### Materials and Substrates

Phenyl‐C_61_‐butyric acid methyl ester (PCBM) was purchased from Nano‐C and Solenne for spin coated and wire‐bar coated samples, respectively. Poly[(5,6‐difluoro‐2,1,3‐benzothiadiazol‐4,7‐diyl)‐alt‐(3,3′′′‐di(2‐octyldodecyl)‐2,2′;5′,2′′;5′′,2′′′‐quaterthiophen‐5,5′′′‐diyl)] (PCE11), with a number‐average molecular mass, *M*_n_ = 83 000 g mol^−1^ and a dispersity *D* ≈ 2.15 was purchased from Ossila. Poly(3‐hexylthiophene‐2,5‐diyl) (P3HT) was purchased from Rieke Metals. 3,9‐bis(2‐methylene‐(3‐(1,1‐dicyanomethylene)‐indanone))‐5,5,11,11‐tetrakis(4‐hexylphenyl)‐dithieno[2,3‐d:2′,3′‐d′]‐s‐indaceno[1,2‐b:5,6‐b′]dithiophene (ITIC) was purchased from 1‐Material. 1,2‐dichlorobenzene (DCB) and chlorobenzene (CB) anhydrous solvents were purchased for Sigma‐Aldrich. Diethylzinc (DEZ) packaged for use in the ALD deposition chamber was purchased from Sigma‐Aldrich. All materials were used as received. A ZnO nanoparticle solution for hole blocking layers (Nanograde N10) was purchased from Nanograde, Switzerland. Prior to deposition, the nanoparticle solution was sonicated for 5 min and filtered through a 0.45 µm PTFE filter. Silicon and indium tin oxide (ITO) covered glass substrates were used for cross section HRSEM and TEM tomography and device characterization, respectively. All substrates were cleaned by sonication in acetone, methanol, and isopropanol for 20 min each. Before device fabrication, the ITO substrates were treated with UV‐ozone for 15 min.

##### Differential Scanning Calorimetry

DSC was performed with a Mettler Toledo DSC700 under N_2_ atmosphere and applying a scan rate of 10 °C min^−1^. Tested powders were produced by drop casting neat components and blend solutions of (5 wt% total material content) in DCB onto glass slides at 100 °C. After solvent evaporation at ambient pressure, films were annealed in vacuum for 7–8 h before scraping them from the substrates as flakes/powders (1.5–4 mg) and sealing them for testing in aluminum crucibles. In the present work, all the shown DSC curves have been normalized by sample mass. A polynomial baseline was subtracted before applying a Gaussian fit to the peaks in order to extract the enthalpy of melting/fusion.

##### Vapor Phase Infiltration

Prior to the VPI procedure, samples were held in a vacuum chamber for 8 h under ≈10^−4 ^ Pa for out‐gassing of remaining solvent. The VPI procedure was carried out in an Ultratech/Cambridge Nanotech Savannah S200 system. Deposition was conducted at 60 °C using 80 alternating cycles of DEZ and water. Each cycle comprised two pulses of the same precursor followed by 20 s hold and 25 s N_2_ purge time for each pulse, four pulses per cycle overall. During the hold time the evacuation valve was closed and the carrier gas flow reduced to 5 sccm. DEZ and water pulse durations were 0.02 and 0.04 s, respectively. The full VPI cycle is illustrated in detail in Figure S2, Supporting Information.

##### High‐Resolution Scanning Electron Microscopy

For cross section HRSEM imaging, the films on silicon substrates were cleaved after immersion in liquid nitrogen. HRSEM micrographs were acquired using a Zeiss Ultra‐Plus FEG‐SEM operated at 2 kV accelerating voltage with a working distance of 2.7 mm.

##### Transient‐Absorption Spectroscopy

TAS measurements were performed using a set up described in more detail elsewhere (see ref. [30]) that was based on an ultrafast laser system (Pharos—Light Conversion, Lithuania) emitting pulses of ≈220 fs at 100 kHz. The pump beam used for these experiments was tuned at 699 nm, while the probe beam extended from 500 to ≈900 nm. Measurements were performed under vacuum conditions, with fluences in the range 0.3–4 µJ cm^−2^. Decay curves at 820 nm were fitted with an asymptotic power law function of the form *y*  =  1/(1 + (*t*/*τ*)^*α*^), where free parameters were *α* and *τ*. Data were considered from 5 ps onward to discard the influence of fast dynamics and instrumental response function.

##### Device and Film Fabrication

Hole blocking layers were deposited onto ITO from a ZnO nanoparticles solution by spin coating at 262 rad s^−1^ (2500 rpm) for 60 s, followed by thermal annealing at 120 °C for 5 min at ambient conditions. The active layers were deposited either by spin‐coating or by wire‐bar coating from solutions of different blend compositions in DCB. The necessary quantities of PCE11, PCBM, and DCB were calculated to achieve 30 mg mL^−1^ total concentration with 45, 55, 80, and 90 wt% of PCE11. For 30 wt%, solutions with total concentration of 50 mg mL^−1^ were prepared. All solutions were stirred at 130 °C for 3 h in N_2_ atmosphere to completely dissolve the polymer. For spin coating, all substrates (Silicon and ITO coated with ZnO NP) and the spin‐coater chuck were heated prior to deposition. To achieve films of a thickness of 300 nm, PCE11:PCBM solutions were spin coated at 1700, 800, 1000, 1350, and 1500 rpm for 20 s followed by 419 rad s^−1^ (4000 rpm) for 2 s, for 30, 45, 55, 80, and 90 wt% PCE11, respectively. After film deposition, samples were left over night in petri dishes inside N_2_ filled glovebox for drying.

The wire‐bar coated films were fabricated using a K‐101 coater (Printcoat Instruments by RKPrint) connected to a temperature‐controlled stage onto which the substrates (glass or Si) were directly placed. In this work, solutions were directly deposited onto the bar, allowing a gap of around 150 µm in between the bar and the substrate. The stage temperature was set at 120 °C while the coating speed was set to 14 cm s^−1^.

To complete device fabrication, the ITO/ZnO NP/PCE11:PCBM samples were transferred to the vacuum chamber for thermal evaporation of the hole‐transporting MoO_3_ interlayer and Al electrode. Thermal evaporation of ≈20 nm MoO_3_ and ≈100 nm Al was conducted through a shadow mask at rates of 0.01 and 0.1 nm s^−1^, respectively, defining an effective device area of 0.03 cm^2^. During evaporation, the system pressure was held at ≈1.3 × 10^−4 ^Pa (10^−6 ^Torr). The solar cells were characterized using a 100 mW cm^−2^ AM 1.5G Newport Inc. solar simulator under N_2_ atmosphere. The *J*–*V* characteristics were measured with a Keithley 2400 source meter. The photovoltaic performances reported in this manuscript were averaged over at least 11 devices of each type.

The ITIC/P3HT bi‐layer was prepared by transferring a P3HT film delaminated from an Si/PEDOT:PSS onto a ITIC film spin coated on Si.

##### Grazing‐Incidence Small‐Angle X‐Ray Scattering

GISAXS was recorded at the CMS beamline at NSLS‐II using 12 keV photons at an angle of incidence with respect to the surface plane of 0.11°. The beam size was adjusted to 200 µm horizontal by 50 µm vertical. SAXS data were collected using a pixel‐array detector (Dectris Pilatus 2M) positioned 2.830 m downstream of the sample; Conversion to q‐space was performed using a silver behenate standard for calibration. A constant air scatter background, obtained by fitting the high q limit, was removed from all data. In‐plane cuts were performed at q*_z_* corresponding to the grazing‐incidence enhanced Yondea peak. Analysis was performed with the Nika package in Igor Pro (Wavemetrics Inc., http://www.wavemetrics.com).^[^ ^31^ ^]^

##### Transmission and Scanning Electron Microscopy/Tomography

Samples for TEM and STEM tomography, 200 and 100 nm thick, respectively, were prepared using FEI Helios NanoLab DualBeam focused ion beam (FIB). TEM and STEM tomography were performed using an FEI Talos 200C FEG‐TEM, operated at 200 kV. For wire‐bar coated samples of 30 wt% PCE11, a series of BF TEM images were acquired at tilt angles ranging from −66° to +64° at angular interval of 2° from −50° to +50° and at angular interval of 1.5° from +50° to +64° and from −50° to −66°. For spin‐coated samples of 45 wt% PCE11, a series of STEM images were acquired using HAADF detector and 300 mm camera length at tilt angles ranging from −63° to +63° at angular interval of 3° from −45° to +45° and at angular interval of 2° from +45° to +63° and from −45° to −63°. Using both TEM and STEM demonstrated the versatility of VPI‐processes films for 3D‐morphology reconstruction.

##### Tomography Reconstructions

Reconstruction was performed on 1400 nm × 200 nm × 340 nm segment of films of 45 wt% PCE11 prepared by spin coating; and on 850 nm × 100 nm × 110 nm segment of a film of 30 wt% PCE11, prepared by wire‐bar coating. For tomography image reconstruction the original image stack was aligned automatically through calculating the 2D normalized cross‐correlation number between two adjacent images in the stack. The pixels that did not represent the materials in the thin film were excluded in the cross‐correlation calculation to maximize the quality of automatic image alignment. Subsequent refining manual alignment was conducted on the images which were of high tilt angles and exhibited large shift of vison on the area of interest, in which case the automatic alignment might fail. The aligned stack was processed through wiener image filter for two iterations to remove background noise. With the de‐noised and aligned tilt series, the 3D reconstruction of the thin film was acquired by using simultaneous iterative reconstruction technique (SIRT) for 50 iterations.^[^ ^32^ ^]^ The raw reconstruction was treated with 3D nonlinear anisotropic diffusion (NAD) filtering for preparation for subsequent segmentation.^[^ ^33^ ^]^ Unlike regular image filtering methods such as Gaussian filter or median filter, the NAD filter smoothed the reconstruction but still preserved the information of the edge between two domains of distinct intensities, which was critical for discerning different phases in this study. The segmentation and visualization of the smoothed reconstruction were performed using FEI Amira software package.

Certain commercial equipment, instruments, or materials (or suppliers, or software, etc.) were identified in this paper to foster understanding. Such identification did not imply recommendation or endorsement by the National Institute of Standards and Technology, nor did it imply that the materials or equipment identified were necessarily the best available for the purpose.

## Conflict of Interest

The authors declare no conflict of interest.

## Supporting information

Supporting Information

Movie1

Movie2

## References

[1] M. A. Ruderer , P. Müller‐Buschbaum , Soft Matter 2011, 7, 5482.

[2] B. Fan , D. Zhang , M. Li , W. Zhong , Z. Zeng , L. Ying , F. Huang , Y. Cao , Sci. China: Chem. 2019, 62, 746.

[3] R. Xue , J. Zhang , Y. Li , Y. Li , Small 2018, 14, 1801793. 10.1002/smll.201801793 30106505

[4] H. Lee , C. Park , D. H. Sin , J. H. Park , K. Cho , Adv. Mater. 2018, 30, 1800453. 10.1002/adma.201800453 29921007

[5] X. Jiao , L. Ye , H. Ade , Adv. Energy Mater. 2017, 7, 1700084.

[6] Y. Huang , E. J. Kramer , A. J. Heeger , G. C. Bazan , Chem. Rev. 2014, 114, 7006. 24869423 10.1021/cr400353v

[7] S. Mukherjee , X. Jiao , H. Ade , Adv. Energy Mater. 2016, 6, 1600699.

[8] N. Majeed , M. Saladina , M. Krompiec , S. Greedy , C. Deibel , R. C. I. MacKenzie , Adv. Funct. Mater. 2020, 30, 1907259.

[9] A. Sánchez‐Díaz , X. Rodríguez‐Martínez , L. Córcoles‐Guija , G. Mora‐Martín , M. Campoy‐Quiles , Adv. Electron. Mater. 2018, 4, 1700477.

[10] A. Harillo‐Baños , X. Rodríguez‐Martínez , M. Campoy‐Quiles , Adv. Energy Mater. 2020, 10, 1902417.

[11] Y. Liu , J. Zhao , Z. Li , C. Mu , W. Ma , H. Hu , K. Jiang , H. Lin , H. Ade , H. Yan , Nat. Commun. 2014, 5, 5293. 25382026 10.1038/ncomms6293 PMC4242436

[12] C. Müller , T. A. M. Ferenczi , M. Campoy‐Quiles , J. M. Frost , D. D. C. Bradley , P. Smith , N. Stingelin‐Stutzmann , J. Nelson , Adv. Mater. 2008, 20, 3510.

[13] P. Wolfer , P. E. Schwenn , A. K. Pandey , Y. Fang , N. Stingelin , P. L. Burn , P. Meredith , J. Mater. Chem. A 2013, 1, 5989.

[14] S. Obuchovsky , B. Shamieh , I. Deckman , G. Ankonina , G. L. Frey , Sol. Energy Mater. Sol. Cells 2015, 143, 280.

[15] S. Obuchovsky , M. Levin , A. Levitsky , G. L. Frey , Org. Electron. 2017, 49, 234.

[16] S. Obuchovsky , H. Frankenstein , J. Vinokur , A. K. Hailey , Y. L. Loo , G. L. Frey , Chem. Mater. 2016, 28, 2668.

[17] C. Z. Leng , M. D. Losego , Phys. Chem. Chem. Phys. 2018, 20, 21506. 30091762 10.1039/c8cp04135k

[18] C. Z. Leng , M. D. Losego , Mater. Horiz. 2017, 4, 747.

[19] J. Guo , H. Ohkita , H. Benten , S. Ito , J. Am. Chem. Soc. 2009, 131, 16869. 19886624 10.1021/ja906621a

[20] C. Baleizão , M. N. Berberan‐Santos , J. Chem. Phys. 2007, 126, 204510. 17552781 10.1063/1.2734974

[21] M. N. Berberan‐Santos , J. Math. Chem. 2005, 38, 165.

[22] N. Li , J. D. Perea , T. Kassar , M. Richter , T. Heumueller , G. J. Matt , Y. Hou , N. S. Güldal , H. Chen , S. Chen , S. Langner , M. Berlinghof , T. Unruh , C. J. Brabec , Nat. Commun. 2017, 8, 14541. 28224984 10.1038/ncomms14541 PMC5322537

[23] E. Buchaca‐Domingo , A. J. Ferguson , F. C. Jamieson , T. McCarthy‐Ward , S. Shoaee , J. R. Tumbleston , O. G. Reid , L. Yu , M. B. Madec , M. Pfannmöller , F. Hermerschmidt , R. R. Schröder , S. E. Watkins , N. Kopidakis , G. Portale , A. Amassian , M. Heeney , H. Ade , G. Rumbles , J. R. Durrant , N. Stingelin , Mater. Horiz. 2014, 1, 270.

[24] P. Westacott , J. R. Tumbleston , S. Shoaee , S. Fearn , J. H. Bannock , J. B. Gilchrist , S. Heutz , J. Demello , M. Heeney , H. Ade , J. Durrant , D. S. McPhail , N. Stingelin , Energy Environ. Sci. 2013, 6, 2756.

[25] P. Westacott , N. D. Treat , J. Martin , J. H. Bannock , J. C. De Mello , M. Chabinyc , A. B. Sieval , J. J. Michels , N. Stingelin , J. Mater. Chem. A 2017, 5, 2689.

[26] F. C. Jamieson , E. B. Domingo , T. McCarthy‐Ward , M. Heeney , N. Stingelin , J. R. Durrant , Chem. Sci. 2012, 3, 485.

[27] N. D. Treat , J. A. N. Malik , O. Reid , L. Yu , C. G. Shuttle , G. Rumbles , C. J. Hawker , M. L. Chabinyc , P. Smith , N. Stingelin , Nat. Mater. 2013, 12, 628. 23727949 10.1038/nmat3655

[28] A. Sharenko , N. D. Treat , J. A. Love , M. F. Toney , N. Stingelin , T. Q. Nguyen , J. Mater. Chem. A 2014, 2, 15717.

[29] H. W. Ro , J. M. Downing , S. Engmann , A. A. Herzing , D. M. Delongchamp , L. J. Richter , S. Mukherjee , H. Ade , M. Abdelsamie , L. K. Jagadamma , A. Amassian , Y. Liu , H. Yan , Energy Environ. Sci. 2016, 9, 2835. PMC7450673 32863865

[30] F. Thouin , D. A. Valverde‐Chávez , C. Quarti , D. Cortecchia , I. Bargigia , D. Beljonne , A. Petrozza , C. Silva , A. R. S. Kandada , Nat. Mater. 2019, 18, 349. 30643234 10.1038/s41563-018-0262-7

[31] J. Ilavsky , J. Appl. Crystallogr. 2012, 45, 324.

[32] P. Gilbert , J. Theor. Biol. 1972, 36, 105. 5070894 10.1016/0022-5193(72)90180-4

[33] A. S. Frangakis , R. Hegerl , J. Struct. Biol. 2001, 135, 239. 11722164 10.1006/jsbi.2001.4406

