# Supplementary material for: Toward Fast Screening of Organic Solar Cell Blends
Source: Adv Sci (Weinh). 2020 Jun 18;7(15):2000960. doi: 10.1002/advs.202000960 (PMC7404169; doi:10.1002/advs.202000960)
Supplement: Supplementary file 1 — Supporting Information [file ADVS-7-2000960-s001.pdf]

© 2020 Wiley-VCH GmbH

ADVANCED SCIENCE
Open Access

Supporting Information

for *Adv. Sci.*, DOI: 10.1002/advs. 202000960

Towards fast screening of organic solar cell blends

Artem Levitsky, Giovanni Maria Matrone, Aditi Khirbat, Ilaria Bargigia, Xiaolei Chu, Oded Nahor, Tamar Segal-Perez, Adam J. Moulé, Lee J. Richter, Carlos Silva, Natalie Stingelin and Gitti L. Frey**

WILEY-VCH

Copyright WILEY-VCH Verlag GmbH & Co. KGaA, 69469 Weinheim, Germany, 2018.

Supporting Information

Towards fast screening of organic solar cell blends

Artem Levitsky, Giovanni Maria Matrone, Aditi Khirbat, Ilaria Bargigia, Xiaolei Chu, Oded Nahor, Tamar Segal-Perez, Adam J. Moulé, Lee J. Richter, Carlos Silva, Natalie Stingelin and Gitti L. Frey**

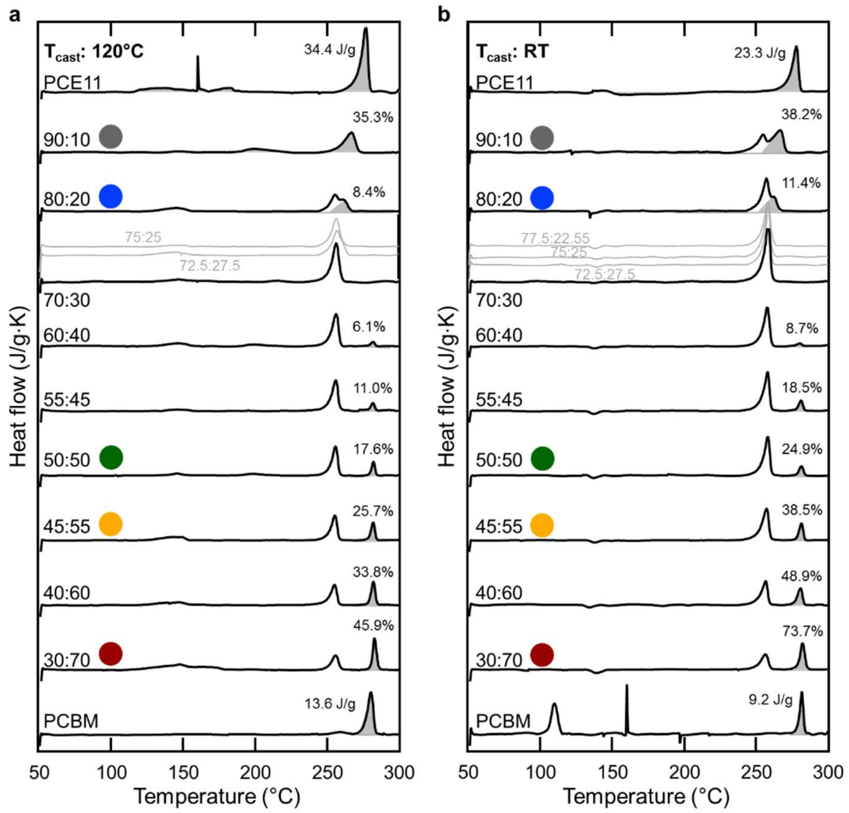

Figure S1. Thermal behaviour of PCE11:PCBM blends. Differential scanning calorimetry (DSC) first heating thermograms of PCE11:PCBM blends, performed at 10 °C/min scan rate in N_2 atmosphere, on films drop-cast at a) 120 °C and b) room temperature. In both scenarios, neat PCE11 melts at ≈282 °C; with a notable melting-point depression being observed upon addition of the fullerene. In contrast, the melting of PCBM (around 285 °C) is little affected by blending with the polymer. The endotherms observed around 258 °C is the eutectic temperature. The deposition conditions, however, do affect the solid-state structure formation and the development of the different phases in the final bulk heterojunction (BHJ). For example, the enthalpy of fusion for neat PCBM, ΔH_f(PCBM), is 13.6 J g^{-1} and 9.2 J g^{-1} and for PCE11, ΔH_f(PCE11), is 34.4 J g^{-1} and 23.3 J g^{-1}, for films drop-cast at 120 °C and at room temperature, respectively. The enthalpies of fusion for the fullerene and the polymer in each blend relative to the respective neat component, $\Delta H_f^{relative}$, are also given.

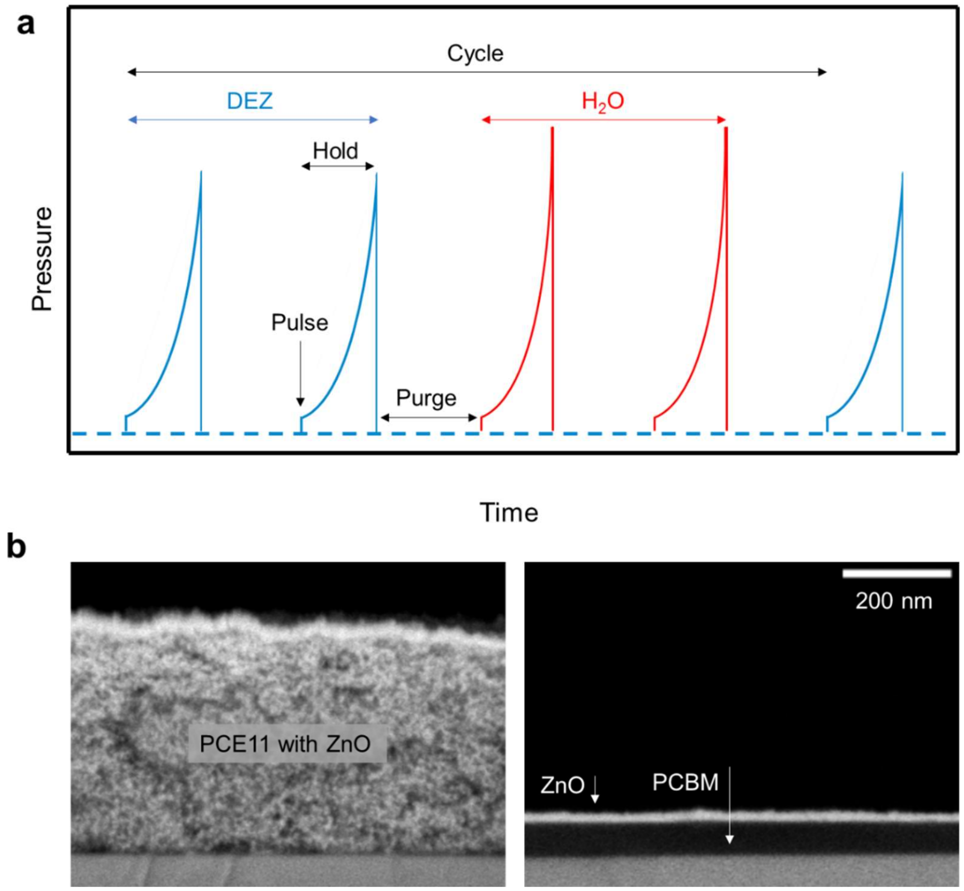

Figure S2. Illustration of the vapor-phase infiltration process targeted for the visualization of BHJ phase morphologies. a) Schematic illustration of the vapor phase infiltration (VPI) cycles used here. b) Cross-section high-resolution scanning electron microscopy (HRSEM) micrographs of neat films of PCE11 (left) and PCBM (right), deposited by spin coating on Si substrates after 80 cycles of VPI at 60 °C. The images were acquired using a backscattered electrons detector (BSE) so that the bright regions correspond to ZnO-rich domains and the dark regions are neat organic domains. The scale bar (200 nm) is for both micrographs

In the VPI process, gaseous precursors generally used for atomic layer deposition (ALD) diffuse through the free volume of an organic matrix and sub-surface deposit inside the organic matrix as shown in Fig. S2. Differentiation between phases is possible when precursor diffusion and retainment occurs in one phase only. Here, diethyl zinc (DEZ) and H_2O gaseous precursors were used to produce ZnO in PCE11:PCBM films, according to the following reaction:

$$\text{Zn-OH}^* + \text{Zn}\left(\text{CH}_2\text{CH}_3\right)_2 \rightarrow \text{Zn-O-Zn-CH}_2\text{CH}_3{}^* + \text{CH}_3\text{CH}_3$$
$$\text{Zn-CH}_2\text{CH}_3{}^* + \text{H}_2\text{O} \rightarrow \text{Zn-OH}^* + \text{CH}_3\text{CH}_3$$

The selective deposition of ZnO in one phase (here the polymer and polymer-rich phase) provides distinct contrast for simple imaging of the domain sizes and the spatial distribution of phases using electron microscopy.

To ensure "staining" of one phase only, we used a VPI process with multiple sequences of prolonged precursor exposure times (Fig. S2a). The images in Fig. S2b show a significant amount of ZnO deposited inside the PCE11 film (bright contrast) due to the diffusion of the precursors into the PCE11 and *in-situ* conversion to ZnO. In contrast, the PCBM film is impermeable leading to no sub-surface deposition of ZnO in the film, but a continuous ZnO layer on the film surface. Even for prolonged exposure times, PCBM remains impenetrable to the gaseous precursors and ZnO is deposited only on the film surface. This selective deposition of ZnO in PCE11 was implemented in this work to effectively map the phase separation in PCE11:PCBM blend films. The VPI process was performed at low temperature (60 °C) to prevent morphology transition during the process. For more details on VPI deposition see ref. [1–5]. [Note: HRSEM is here used to help visualize the effect of VPI on blend films. We do not regard it as an independent tool that by itself can provide useful information on BHJs.]

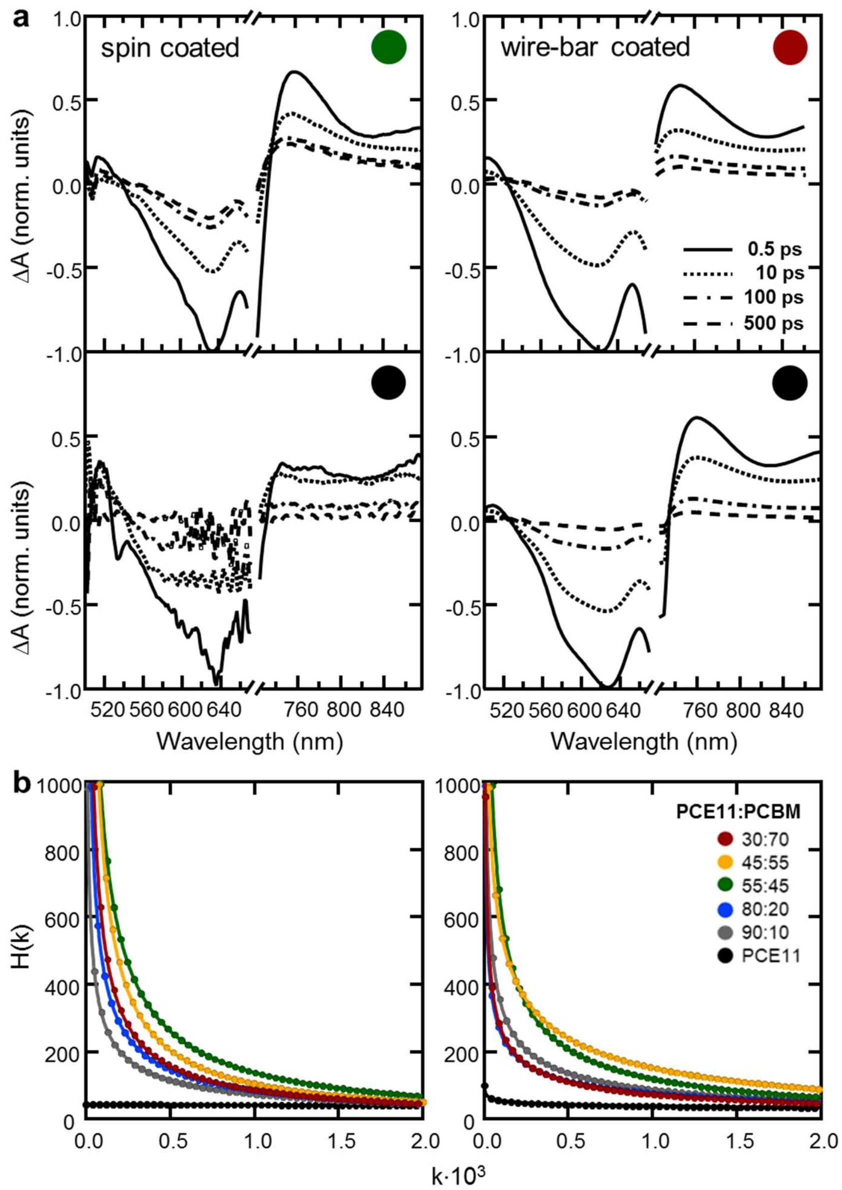

Figure S3. Transient absorption spectra and rate constants distribution of PCE11:PCBM blends. a) Transient absorption spectra as a function of pump-probe delay of spin coated 55 % mass fraction (wt%) PCE11 and neat polymer films (left column top and bottom, respectively); and wire-bar coated 30 wt% PCE11 and neat polymer films (right column top and bottom, respectively) films. All data are normalized at the value of the minimum differential absorbance (ΔA) signal for the curve at 0.5 ps. These spectra show a ground state bleaching signal in the (500 to 690) nm range and a photo-induced absorption feature at (720 to 890) nm that, by comparison to previous studies, can be ascribed to the absorption of polrons photo-generated by the pump pulse.[6] b) Rate constants distribution for spin-coated (left) and wire-bar coated (right) films. These distributions have been derived by Laplace transform the asymptotic power law functions with α and τ_0 parameters derived by fitting transient absorption data at 820 nm.

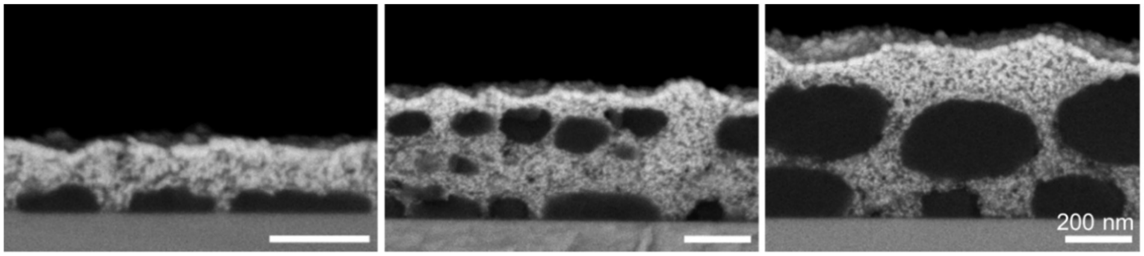

Figure S4. Phase morphology evolution with film thickness of wire-bar coated films. Cross-section HRSEM BSE micrographs of wire-bar coated PCE11:PCBM blend films of 45 wt% PCE11 content after a VPI process of exposure to 80 cycles of DEZ and water at 60 °C. Film thickness measured from HRSEM micrographs from left to right are 130 nm, 380 nm and 500 nm. ZnO deposition inside the films clearly reveals the blends' phase morphology evolution from discontinuous bilayer-like morphology for thin films to morphology reminiscent for structures formed via liquid-liquid phase separation, characterized by sphere-like, coarse PCBM rich domains. The scale bar for all the micrographs is 200 nm.

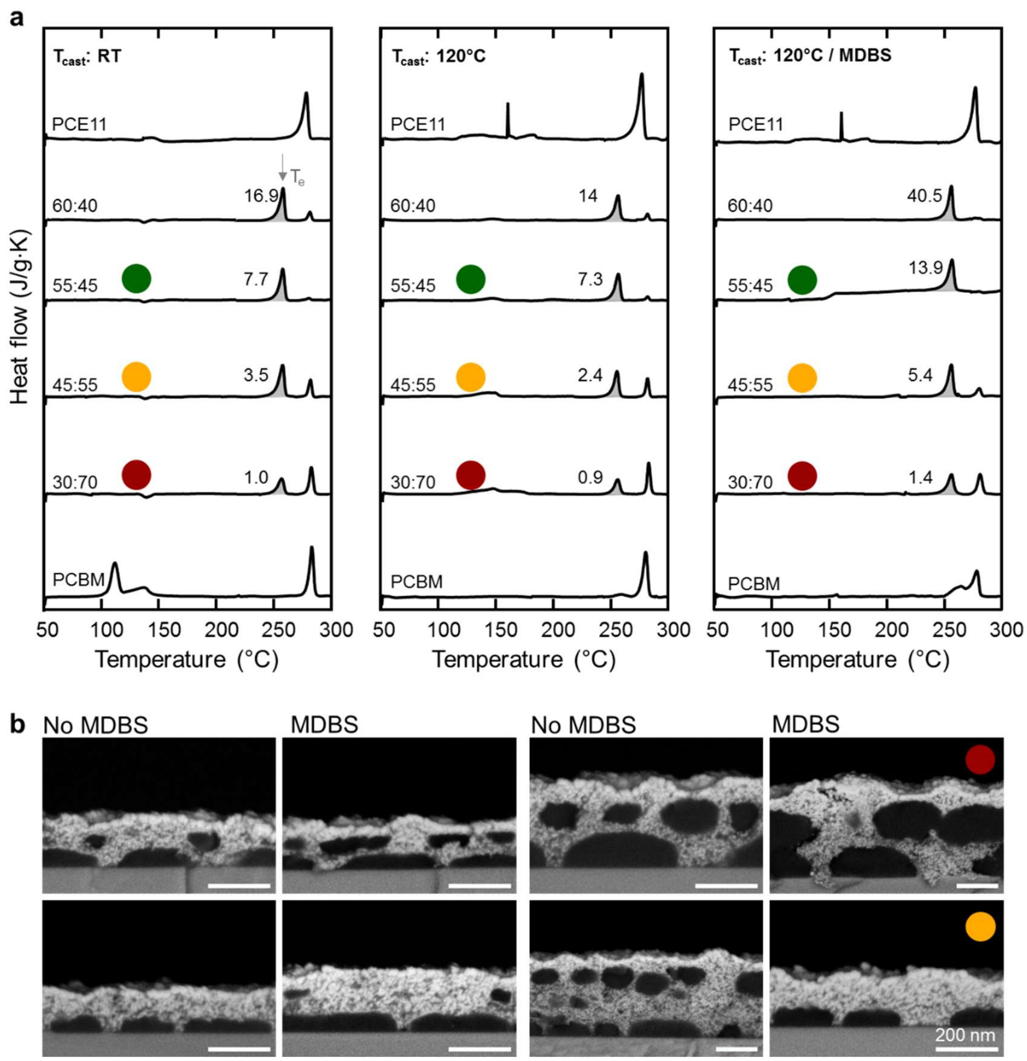

Figure S5. Effect of addition of nucleating agent 1,3:2,4-di-o-methylbenzylidene-*D*-sorbitol (MDBS) on the thermal behaviour and phase morphology of PCE11:PCBM blends. a) Differential scanning calorimetry (DSC) first heating thermograms of PCE11:PCBM blends, performed at 10 °C min^{-1} scan rate in N_2 atmosphere on films drop-cast at room temperature (left), 120 °C (middle) and 120 °C with the addition of the nucleating agent MDBS (right). The values given in the graphs are the ratio of the enthalpy of the eutectic temperature divided by the enthalpy of the fullerene melting transition (see Table S1 for a summary of the enthalpy values). b) Cross-section HRSEM BSE micrographs of wire-bar coated PCE11:PCBM films of two fullerene-rich blend compositions, after exposure to DEZ and water, showing selective deposition of ZnO in the polymer-rich domains. Films were fabricated with and without the addition of the nucleating agent MDBS. The four left panels refer to thin films, while the four right panels refer to thicker films. The scale bar for all the micrographs is 200 nm.

Table S1. Effect of addition of nucleating agents on the molecular order of eutectic domains vs. pro-eutectic fullerene domains deduced from thermal analysis. Enthalpies of fusion for the eutectic (EU) temperature and the PCBM melting, and their ratio, obtained for PCE11:PCBM blends cast at different temperatures with and without addition of the nucleating agent MDBS. The values were deduced by fitting the endotherms of the respective transitions with simple Gaussians after the definition of a straight line as base-line.

**Blend Composition**	**RT**			**120 °C**			**MDBS**		
	ΔH_f^{EU} [J g^{-1}]	ΔH_f^{PCBM} [J g^{-1}]	ratio	ΔH_f^{EU} [J g^{-1}]	ΔH_f^{PCBM} [J g^{-1}]	ratio	ΔH_f^{EU} [J g^{-1}]	ΔH_f^{PCBM} [J g^{-1}]	ratio
30:70	6.5	6.8	1	5.3	6.2	0.9	8.0	5.6	1.4
40:60	8.9	4.5	2	6.5	4.6	1.5			
45:55	12.2	3.5	3.5	8.5	3.5	2.4	11.9	2.2	5.4
50:50	13.1	2.3	6	9.7	2.4	4			
55:45	13.1	1.7	7.7	11.0	1.5	7.3	13.9	1.0	13.9
60:40	13.5	0.8	17	11.2	0.8	14	16.2	0.4	40.5

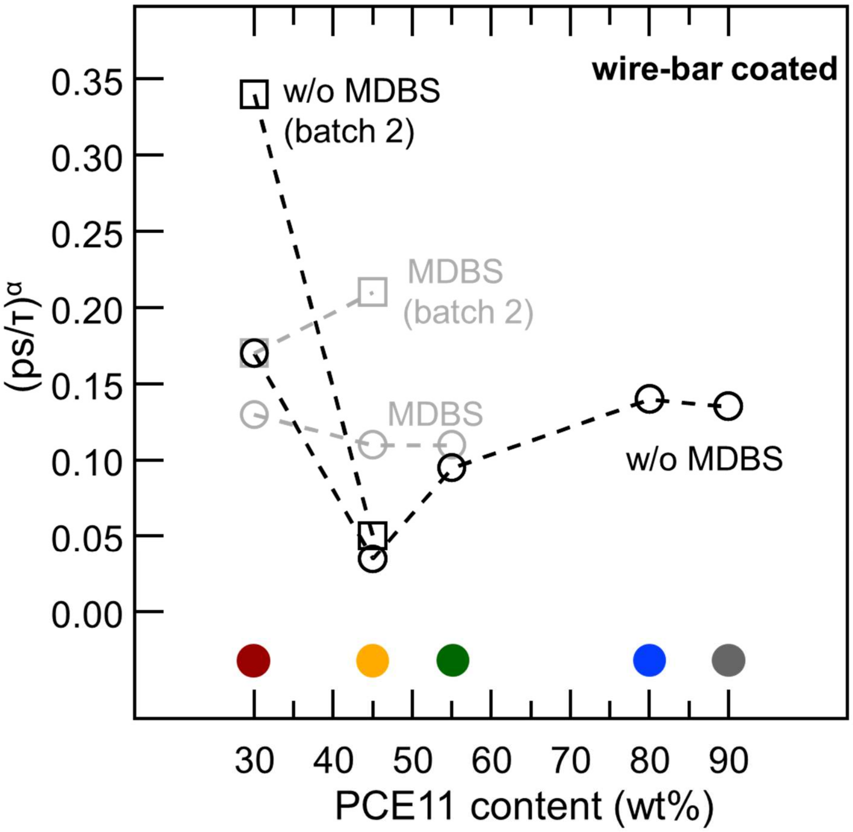

Figure S6. Effect of addition of nucleating agent on the charge-carrier population dynamics in PCE11:PCBM blends. Addition of the nucleation agent strongly affects the transient absorption dynamics, reducing the composition dependence, however, at the expense of a generally faster decay comparable to the decay measured for pristine PCE11-rich blends.

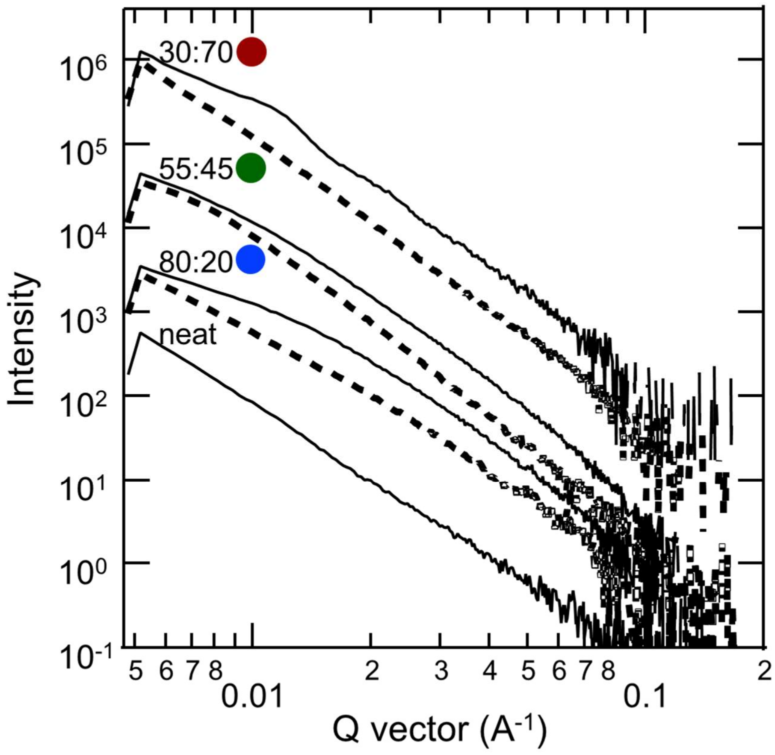

Figure S7. GISAXS profiles for spin-coated neat PCE11 films and PCE11:PCBM blends, wire-bar coated (dotted lines) and spin-coated (solid lines). The spin-coated films generally exhibited more scattering than the wire-bar coated architectures, indicating that the deposition techniques affect the films' phase morphology. Only the spin-coated films comprising 30 wt% PCE11 showed a distinct feature at ≈0.012 $Å^{-1}$ indicating that these samples have characteristic domain sizes of ≈52 nm. In all the other cases, a precise domain size cannot be extracted either because the phase separation is too fine (e.g. in the samples of high PCE11 content) or too rough (e.g., wire-bar coated films of high PCBM content).

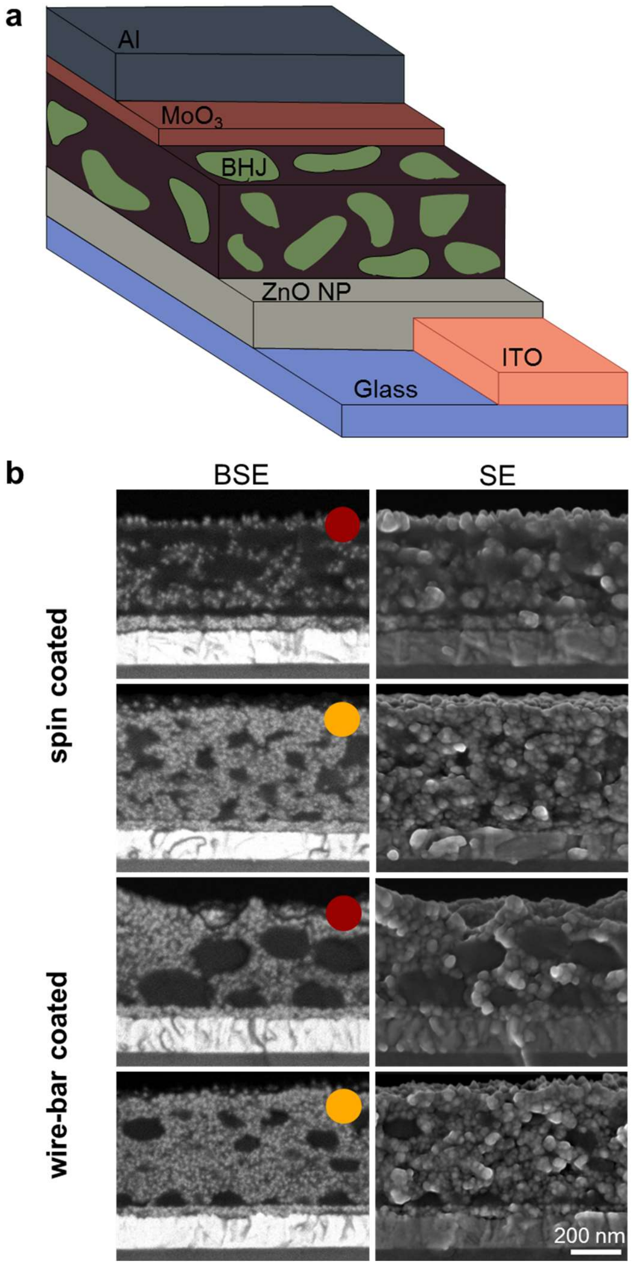

Figure S8. VPI "staining" of complete OSC showing the solid state microstructure of PCE11:PCBM BHJ. a) Schematic of the device structure used in this work: ITO/ZnO nano-particles/PCE11:PCBM BHJ/MoO_3/Al. b) Cross-section HRSEM BSE micrographs (left column) and secondary electrons signal (SE, right column) of spin-coated (top row) and wire-bar coated (bottom row) PCE11:PCBM films of two compositions: 30 wt% PCE11 (red dot) and 45 wt% PCE11 (yellow dot) after VPI. The VPI "staining" process was done by exposing the OSCs to 80 cycles of DEZ and water at 60 °C. The precursors diffuse into the films through areas between the patterned electrodes where the BHJ surface is exposed and selectively deposit ZnO in the PCE11-rich domains (bright contrast). The morphology of the BHJ in the OSC devices are in agreement with those obtained for films of the same compositions (Figure 1c) corroborating the usefulness of VPI for the fast screening of OSCs. The scale bar for all the micrographs is 200 nm.

Supportin Information Movies (in separate files):

Movie 1: 3D-morphology reconstruction of the 45 wt% PCE11 film prepared by spin coating.

Movie 2: 3D-morphology reconstruction of the 30 wt% PCE11 film prepared by wire-bar coating.

References:

[1] S. Obuchovsky, H. Frankenstein, J. Vinokur, A. K. Hailey, Y. L. Loo, G. L. Frey, *Chem. Mater.* **2016**, *28*, 2668.

[2] S. Obuchovsky, B. Shamieh, I. Deckman, G. Ankonina, G. L. Frey, *Sol. Energy Mater. Sol. Cells* **2015**, *143*, 280.

[3] S. Obuchovsky, M. Levin, A. Levitsky, G. L. Frey, *Org. Electron.* **2017**, *49*, 234.

[4] C. Z. Leng, M. D. Losego, *Mater. Horizons* **2017**, *4*, 747.

[5] C. Z. Leng, M. D. Losego, *Phys. Chem. Chem. Phys.* **2018**, *20*, 21506.

[6] J. Guo, H. Ohkita, H. Benten, S. Ito, *J. Am. Chem. Soc.* **2009**, *131*, 16869.
